# Dyslipidemia in Adolescents Seen in a University Hospital in the city of Rio de Janeiro/Brazil: Prevalence and Association

**DOI:** 10.5935/abc.20180254

**Published:** 2019-02

**Authors:** Nathalia Pereira Vizentin, Paula Mendonça Santos Cardoso, Camila Aparecida Gomes Maia, Isabela Perez Alves, Gabriel Lunardi Aranha, Denise Tavares Giannini

**Affiliations:** 1 Núcleo de Estudos da Saúde do Adolescente do Hospital Universitário Pedro Ernesto, Rio de Janeiro, RJ - Brazil; 2 Hospital Maternidade Therezinha de Jesus da Faculdade de Ciências Médicas e da Saúde de Juiz de Fora, Juiz de Fora, MG - Brazil

**Keywords:** Hyperlipidemias, Adolescent, Obesity, Sedentary Lifestyle, Anthropometry, Cardiovascular Diseases, Risk Factors

## Abstract

**Background:**

Early exposure to obesity favors greater risks of cardiovascular factors such as dyslipidemia.

**Objectives:**

To establish the prevalence of dyslipidemia, and to evaluate its association with nutritional status of the adolescents attended at the ambulatory of the Adolescent Health Studies Center of the University Hospital Pedro Ernesto.

**Methods:**

This is a cross-sectional, observational study, the sample of which was of convenience, consisting of adolescents from 12 to 18 years old of both genders. The lipid profile was evaluated, along with its association with the anthropometric indicators: body mass index and waist circumference. For statistical analysis, a significance level of 5% was used.

**Results:**

A total of 239 adolescents, 104 boys (43.5%) and 135 girls (56.5%) were evaluated and, of these, 52 (21.8%) were eutrophic, 60 (25.1%) overweight, and 127 (53.1%) obese. Obeseadolescents had significantly lower mean values of HDL-cholesterol (44.7 mg/dl vs 53.9 mg/dl; p < 0.001) and higher triglycerides (109.6 mg/dl vs 87.3 mg/dl; p = 0.01). The changes with higher prevalence were low HDL-cholesterol (50.6%), hypercholesterolemia (35.1%), and hypertriglyceridemia (18.4%). A negative association of HDL-cholesterol with body mass index and a positive association of triglycerides with body mass index could be observed, even after adjustment for gender and skin color.

**Conclusion:**

This study demonstrated high prevalence of dyslipidemia among adolescents. In view of the significant association between lower levels of HDL-cholesterol and increased triglycerides with overweight, the control of these factors should receive attention, with the precocious diagnosis of the dyslipidemia being important, mainly if it is associated with another cardiovascular risk, to develop effective intervention strategies.

## Introduction

Adolescence is a period of intense modification that takes place between childhood and adulthood, and is highlighted by explicit development, growth and body changes. During adolescence there is a physiological increase of the tissues, including adipose tissue, especially in girls, being a critical period to initiate or exacerbate obesity.^1-3^

In the current scenario, low consumption of fruit and vegetables and high consumption of processed food,^3,4^ along with excessive use of electronic devices and low frequency of regular practice of physical activities were observed among adolescents. It is also observed that the omission of meals and the intake of fast food are also common habits in this age group. Such conditions favor weight gain and risk factors for chronic diseases.^2-6^

Data from the Brazilian Institute of Geography and Statistics (IBGE) show a clear increase in the prevalence of overweight and obesity in adolescents in the last 34 years in Brazil, from 1974-1975 to 2008-2009, from 3.7% to 21.7% in boys and 7.6% to 19.4% in girls.^7^ This situation is a concern because obesity is a considerable risk factor for chronic non-communicable diseases, being highlighted among dyslipidemias, which is even more pronounced when associated with a sedentary lifestyle.^8^ Early exposure to obesity favors a higher cardiovascular risk not only in childhood and adolescence, but also a high incidence of premature mortality in adults who were obese in these phases of life.^9,10^ Overweight in childhood and adolescence is considered a more powerful predictor of these risks than overweight in adulthood.^9-11^

Dyslipidemia is understood as changes in the lipid profile, which may occur by the elevation in total cholesterol (TC), LDL-cholesterol (LDL-c), triglycerides (TG), or decrease in HDL-cholesterol (HDL-c), with these being primary (genetic factors) or secondary (environmental factors) causes.^8,12-14^ These changes alone and mainly when accompanied of other risk factors may lead to the development of atherosclerosis.^13^

The present study aimed to establish the prevalence of dyslipidemia and to evaluate its association with the nutritional status of adolescents seen at the secondary care clinic of the University Hospital Pedro Ernesto (HUPE) Center of Adolescent's Health Studies (NESA).

## Methods

This is an observational, cross-sectional study, the sample of which was of convenience, consisting of adolescents aged between 12 and 18 years of age, of both genders, referred internally or through the National Regulation System (SISREG) to the Nutrition service with a diagnosis of overweight, dyslipidemia, glucose metabolism changes, or other comorbidity, being attended at the NESA outpatient clinic. Adolescents with a diagnosis of thinness according to body mass index (BMI)/Age; using drugs that may interfere with laboratory tests (statins, steroids, bile acid sequestrants); or who are followed due to genetic syndromes, nephrotic syndrome, familial hypercholesterolemia, rheumatic diseases, type 1 Diabetes Melittus, hypothyroidism, eating disorder, or disabsorption diseases were excluded.

Demographic data such as age, gender, skin color, and anthropometric data such as weight, height and waist circumference (WC) were collected. The weight (kg) was measured using a Micheliti® electronic digital scale, with an accuracy of 0.1 kg and a maximum of 200 kg, with the adolescent in his/her barefoot, wearing light clothes, and in an orthostatic position.^15^ For height (cm), a Sanny® stadiometer fixed to the wall was used, with a precision of 0.1 cm, with the adolescent in his/her barefoot, and the body in anatomical position, head parallel to the ground according to Frankfurt plane.^15^ Such measurements were used for the assessment of the adolescent’s nutritional status through the BMI for age in z-scores, and the proposal of the World Health Organization for children and adolescents from 5 to 19 years being adopted as reference.^16^

WC measurements were performed using an anthropometric inelastic tape with a 0.1 centimeter scale at the midpoint between the last costal arch and the iliac crest at the end of normal expiration. They were classified according to the proposal by Fernández et al.,^17^ with the WC being elevated when ≥ 75th percentile. The lipid profile evaluation consisted of the following laboratory tests: TC, TG, HDL-c, and LDL-c. To obtain the glucose and lipid profile data, the blood test was always performed with a previous fasting of 12 hours. TG, TC, and HDL-c were measured through the enzymatic colorimetric method, and LDL-c calculated with Friedewald’s formula.^18^ The reference values used were those recommended by the I Guidelines for the prevention of atherosclerosis in childhood and adolescence.^8^

### Statistical analysis

The analyzes were performed using STATA 14 software. The continuous variables were presented as mean and standard deviation and the categorical variables as absolute frequency. The distribution of variables was assessed using the Kolgomorov-Sminorv test. The comparisons of the continuous variables with normal distribution were performed with the unpaired Student’s t-test and for more than two independent groups, one way variance analysis (ANOVA) and Post Hoc test were used. For the comparisons of categorical variables, the chi-square test or Fisher’s exact test was used. For the study of the association, correlation analyzes (Pearson or Spearman) and simple and multiple linear regression were performed. A significance level of 5% was considered in all analyzes. The research project was approved by the Research Ethics Committee of HUPE/UERJ, registry CEP/HUPE: 3051/2011; CAAE: 0193.0.228.000-11.

## Results

A total of 239 adolescents with a mean age of 14.4 ± 1.8 years was evaluated, with 104 boys (43.5%), and 135 girls (56.5%). Table 1 describes the anthropometric characteristics and the lipid profile mean of the population evaluated according to gender. The girls had statistically higher BMI mean values, and HDL-c, while THE mean height was higher in boys.

**Table 1:** Mean and standard deviation of the anthropometric and lipid profiles of the total sample, stratified by gender

Variable			Gender				p value
	Total (n = 239)		Female (n = 135)		Male (n = 104)		
	Mean	SD	Mean	SD	Mean	SD	
Weight (kg)	76.2	± 22.4	74.8	± 22.2	77.9	± 22.7	0.14
Height (cm)	162.8	± 0.1	159.0	± 0.1	167.6	± 0.1	< 0.01*
BMI (kg/m^2^)	28.5	± 7.4	29.4	± 7.8	27.5	± 6.8	0.02*
WC (cm)	89.9	± 15.1	92.3	± 15.9	88.1	± 14.4	0.06
TC (mg/dl)	160.3	± 34.1	163.3	± 34.9	156.5	± 32.9	0.06
LDL-C (mg/dl)	93.9	± 29.2	95.5	± 29.3	92.0	± 29.0	0.18
HDL-C (mg/dl)	47.6	± 14.0	49.4	± 15.4	45.2	± 11.6	0.01*
TG (mg/dl)	99.4	± 53.7	99.1	± 53.8	99.9	± 53.8	0.46

Statistical test: unpaired Student's t test;

* Statistically significant difference (p < 0.05); SD: standard deviation; BMI: body mass index; WC: waist circumference; TC: total cholesterol; LDL-c: low density lipoprotein; HDL-c: high density lipoprotein; TG: triglycerides.

Table 2 describes the anthropometric characteristics and the lipid profile of the population evaluated according to nutritional status. The nutritional status classification revealed that 53.1% of the adolescents were obese, 25.1% overweight, and 21.8% eutrophic. The eutrophic adolescents had mean values of HDL-c significantly higher than the obese ones. Regarding triglycerides, the obese adolescents had values that were significantly higher than the eutrophic ones.

**Table 2:** Mean and standard deviation of anthropometric characteristics and lipid profile according to nutritional status

Variable	Nutritional status according to BMI						p value
	Eutrophy (n = 52)		Overweight (n = 60)		Obesity (n = 127)		
	Mean	SD	Mean	SD	Mean	SD	
Weight (kg)	52.3	± 10.5	66.9	11.7	± 90.3	19.0	< 0.01*
Height (cm)	162.0	± 0.1	162.1	0.1	± 163.3	0.1	0.62
BMI (kg/m^2^)	19.7	± 2.3	25.3	2.1	± 33.7	5.9	< 0.01*
WC (cm)	77.3	± 10.1	82.6	9.8	± 96.5	14.9	< 0.01*
TC (mg/dl)	158.6	± 34.8	159.1	35.6	± 161.6	33.4	0.82
LDL-C (mg/dl)	87.2	± 26.2	94.8	29.1	± 96.3	30.1	0.16
HDL-C (mg/dl)	53.9	± 16.2	48.2	12.6	± 44.7	12.9	< 0.01*
TG (mg/dl)	87.3	± 45.1	88.5	46.2	± 109.6	58.3	0.01*

Statistical test: ANOVA (One Way) and Post Hoc test;

* Statistically significant difference (p < 0.05); BMI: body mass index; WC: waist circumference; CT: total cholesterol; LDL-c: low density lipoprotein; HDL-c : high density lipoprotein; TG: triglycerides.

The most prevalent changes were low HDL-c (50.6%), hypercholesterolemia (35.1%), and hypertriglyceridemia (18.4%). Regarding the prevalence of lipid profile changes, according to gender, it was observed that the girls showed higher prevalence of change, but with no statistically significant difference. The prevalence of lipid profile changes in girls and boys were respectively 64.3% and 35.7% (p = 0.07) for high TC, 73.1% and 26.9% (p = 0.07) in the LDL-c, 50.4% and 49.6% (p = 0.05) in HDL, and 59.1% and 40.6% (p = 0.07) in TG. Table 3 presents the prevalence of changes in lipid profile according to the nutritional status by BMI. The prevalence of low HDL-c was significantly higher (p = 0.01) in obese patients.

**Table 3:** Prevalence of dyslipidemias according to nutritional status by BMI

Lipids	Nutritional diagnosis				p value
	Total (n: 239)	Eutrophy (n: 52)	Overweight (n: 60)	Obesity (n: 127)	
**TC (mg/dl)**					
Normal	155(64.8%)	35 (22.6%)	39 (25.2%)	81 (52.3%)	0.90
Changed	84(35.2%)	17 (20.2%)	21 (25%)	46 (54.8%)	
**LDL-C (mg/dl)**					
Normal	213 (89.1%)	43 (23%)	50 (23.5%)	114 (53.5%)	0.18
Changed	26 (10.9%)	3 (11.5%)	10 (38.5%)	13 (50.0%)	
**HDL-C (mg/dl)**					
Normal	118 (49.4%)	35 (29.7%)	31 (26.3%)	52 (44.0%)	0.01*
Changed	121 (50.6%)	17 (14.0%)	29 (24.0%)	75 (62.0%)	
**TG (mg/dl)**					
Normal	195 (81.6%)	47 (24.1%)	51 (26.2%)	97 (49.7%)	
Changed	44 (18.4%)	5 (11.4%)	9 (20.4%)	30 (68.2%)	0.06

Statistical test: Chi square;

* Statistically significant difference (p < 0.05); TC: total cholesterol; LDL-c: low density lipoprotein; HDL-c: high density lipoprotein; TG: triglycerides.

In this study, a negative correlation was observed between BMI and HDL-c (r = -0.23, p < 0.01) and a positive correlation between BMI (r = 0.25, p < 0.01) and WC (r = 0.20, p = 0.03) with TG. In the bivariate and multivariate linear regression analysis the negative association of BMI with HDL-c was maintained, as well as the positive association of BMI and WC with TG even after adjustment for gender and skin color (Table 4).

**Table 4:** Bivariate and multivariate linear regression analysis between lipid profile and anthropometric variables**

Variables		BMI			WC			p value
	Gross Coef(95% CI)	p value	Adjusted Coef (95% CI)	p value	Gross Coef (95% CI)	p value	Adjusted Coef (95% CI)	
TC (mg/dl)	0.02 (-0.01 - 0.04)	0.19	0.01 (-0.01 - 0.04)	0.33	0.05 (-0.02 - 0.13)	0.17	0.06 (-0.02 - 0.13)	0.13
LDL-c (mg/dl)	0.03 (-0.00 - 0.06)	0.07	0.02 (-0.01 - 0.06)	0.14	0.07 (-0.01 - 0.16)	0.08	0.08 (-0.01 - 0.16)	0.08
HDL-c (mg/dl)	-0.12 (-0.18 - -0.05)	p < 0.01 *	-0.13 (-0.20 - -0.07)	p < 0.01 *	-0.22 (-0.47 - 0.02)	0.07	-0.23 (-0.47 - 0.02)	0.07
TG (mg/dl)	0.03 (0.02 - 0.06)	p < 0.01 *	0.04 (0.02 - 0.05)	p < 0.01 *	0.05 (0.00 - 0.10)	0.03*	0.06 (0.01 - 0.10)	0.02*

Statistical test: Bivariate and multivariate linear regression;

* Statistically significant difference (p < 0.05);

** Adjusted for gender and skin color; BMI: body mass index; WC: waist circumference; COEF: coefficient; CI: confidence interval; TC: total cholesterol; LDL-c: low density lipoprotein; HDL-c: high density lipoprotein; TG: triglyceride.

## Discussion

This study presented higher mean values of the lipid profile than others in the literature.^19-21^ HDL-c, a lipoprotein that acts as a protective factor against cardiovascular diseases, was the component with the highest change prevalence found among adolescents, as well as in the study by Ribas and da Silva, 2009.^22^ Another population-based study with more than 30,000 participants also found similar results.^19^

This fact is extremely worrying, because dyslipidemia alone and mainly accompanied by other factors, either environmental or genetic, can condition the development of atherosclerosis and, consequently, increase the risk of cardiovascular events. It is fundamental to always consider the prevention and treatment of dyslipidemias, from childhood to adolescence, to reduce the risks of cardiovascular diseases.^11,13^

The lipid profile may vary during adolescence, and the female gender usually has higher levels, a fact that may be justified by the menarche.^23^ Although no significant difference between the lipid profile means within the genders was observed, it is possible to notice that the girls had higher values for all parameters, and this is commonly observed in the literature.^19,22,24^

In the study by Garcez MR et al.,^20^ it was observed that overweight adolescents had higher mean values for TC, LDL-c and TG, as well as low HDL-c, as in this study.^20^ Similarly, Oliveira et al.^24^ found such results when they assessed the lipid profile according to the nutritional status.^24^ The main changes that are usually associated with obesity in this age group, and which have been observed as the standard are changes in HDL-c and TG.^25,26^ This study demonstrated an association between HDL-c/TG and WC/BMI, showing the relationship with adiposity. This same association was seen in other studies, such as in the one by Pavão et al.,^21^ when they evaluated adolescents in a municipality of the state of Paraná and observed a predisposition to dyslipidemia when abdominal obesity, seen through WC, was present. Another study in the city of Recife showed that adolescents with overweight or abdominal obesity had higher values of TG and lower levels of HDL-c.^25^ This study had as a limitation a convenience sample, which does not allow the generalization of results.

## Conclusions

The present study demonstrated a high prevalence of dyslipidemia among adolescents seen at NESA outpatient clinic, mainly low HDL-c in obese adolescents. Considering the significant association between low levels of HDL-c and TG increased with adiposity, the control of these factors should receive attention, with the investigation and early diagnosis of the lipid change being important, especially if it is associated with another cardiovascular risk such as obesity, to develop effective intervention strategies. In addition, data presented show an alert to the multiprofessional team about the need for a greater incentive to healthy lifestyle measures in the above-mentioned population.
